# Growth-differentiation factor-8 (GDF-8) in the uterus: its identification and functional significance in the golden hamster

**DOI:** 10.1186/1477-7827-7-134

**Published:** 2009-11-25

**Authors:** Chun Lung Wong, Ya Yu Huang, Wing Kei Ho, Hong Kit Poon, Pui Lai Cheung, Wai Sum O, Pak Ham Chow

**Affiliations:** 1 School of Biomedical Sciences, Faculty of Medicine, the Chinese University of Hong Kong, Shatin, Hong Kong, PR China; 2 Department of Anatomy, Li Ka Shing Faculty of Medicine, the University of Hong Kong, Sassoon Road, Hong Kong, PR China

## Abstract

Transforming growth factor-beta superfamily regulates many aspects of reproduction in the female. We identified a novel member of this family, growth-differentiation factor 8 (GDF-8) in the 72 h post coital uterine fluid of the golden hamster by proteomic techniques. Uterine GDF-8 mRNA decreased as pregnancy progressed while its active protein peaked at 72 h post coitus (hpc) and thereafter stayed at a lower level. At 72 hpc, the GDF-8 transcript was localized to the endometrial epithelium while its protein accumulated in the stroma. Exogenous GDF-8 slowed down proliferation of primary cultures of uterine smooth muscle cells (SMC) and endometrial epithelial cells (EEC). In addition, GDF-8 attenuated the release of LIF (leukaemia inhibiting factor) by EEC. As for the embryo in culture, GDF-8 promoted proliferation of the trophotoderm (TM) and hatching but discouraged attachment. Our study suggests that GDF-8 could regulate the behavior of preimplantation embryos and fine-tune the physiology of uterine environment during pregnancy.

## Background

The TGF-β superfamily encompasses a large number of structurally related molecules such as TGF-β1-3, growth-differentiation factors (GDF), nodal molecules, activins, inhibins and bone morphogenetic proteins (BMP) [1,2]. In reproduction, these molecules function in regulating immune responses in the uterus during embryogenesis and embryonic implantation [2-4]. It has been suggested that some of these molecules can be used as markers to screen various potential gestational problems like miscarriage, placental pathology and pre-eclampsia [2,5,6]. So the emergence of novel members of this family could open up more therapeutic choices. In this study, we reported the identification of a novel member of TGF-β superfamily, GDF-8 in the pregnant uterus and studied its functional significance in the uterus of the golden hamster.

GDF-8, also known as myostatin, was first identified in skeletal muscle [7]. As the name implies, its primary action is on skeletal muscle growth [7,8]. GDF-8 is synthesized as a precursor made up of 375 amino acids and released in an active glycosylated form (26 kDa) into the plasma [9] where it binds to the activin receptor 2B (ACVR2B) to exert its effect [8]. Recently its presence and cyclical expression in the non-pregnant uterus of the rat has been reported [10]. This expression pattern is regulated by steroid hormones [10] but its function is not known.

We detected and localized in the hamster the expression of GDF-8 mRNA and its active peptide in the uterus throughout the course of pregnancy from 60 h*pc *(D2.5) when the embryo started to enter the uterus, to D15 shortly before delivery. In this species, embryos complete the oviduct-uterine transit by 72 h*pc *(D3) in preparation for attachment that begins at 78 h*pc*; implantation follows and is completed on D5 [11,12]. We studied the effect of GDF-8 on proliferation and differentiation of myometrial smooth muscle, endometrial epithelium and development of the blastocyt.

## Methods

All materials were purchased from Sigma (St Louis, MO, USA) unless otherwise stated and all procedures were carried out at room temperature unless specified.

### Experimental animals

Randomly bred golden hamsters (*Mesocricetus auratus*) supplied by and housed in the Laboratory Animal Services Centre of The Faculty of Medicine, The Chinese University of Hong Kong were handled in compliance with a protocol approved by the Animal Research Ethics Committee of the Institution. The hamsters were kept under a photoperiod of 14 hours light: 10 hours darkness (lights on at 11 am). On the day of *estrus*, a 6-8 weeks old female hamster with two consecutive normal cycles according to vaginal discharge was mated for 15 min with a male and thereafter kept individually in a breeding cage. The day of mating was designated day 0 of pregnancy.

### Sample collection

At designated time points after mating, female hamsters were sacrificed with an overdose of ketamine/xylazine. Uterine horns were removed and each was flushed with 1 ml 0.85% saline containing proteinase inhibitors (Roche, Mannheim, Germany). The flushing was centrifuged at 12000 g for 10 min at 4°C and the supernatant kept. The endometrium was then peeled off from the uterus. The flushing and endometrial tissues were stored separately at -80°C. Intact uteri for histological studies were fixed in 4% paraformaldehyde overnight, dehydrated in graded ethanol, embedded in paraffin and processed for immunohistochemistry and *in situ *hybridization (ISH).

### Extraction of protein and RNA

Uterine flushing or endometrial tissue was incubated on ice for 3 h in 1 ml lysis buffer made up of 7 M urea, 65 mM CHAPS (USB Corporation, Cleveland, OH, USA), 2 M thiourea, 1% NP-40, 1% TBP reducing agent (Bio-Rad Laboratories, Hercules, CA, USA) and proteinase inhibitors. The mixture was centrifuged at 12000 g for 20 min at 4°C and the supernatant was kept. Protein concentration was determined with Modified Bradford reagent (Bio-Rad, Hercules, CA, USA). Total RNA was extracted from endometrial tissue by using the Promega SV total RNA isolation kit (Promega, Madison, WI, USA) according to manufacturer's instructions. The RNA extracted was reversely transcribed with Superscript RNase-H Reverse transcriptase (Invitrogen, Carlsbad, CA, USA). The cDNA was stored at -20°C until use.

### 2-Dimensional electrophoresis

Two-dimensional electrophoresis (2-DE) was carried out as described in Liu et al. [13]. One hundred milligram of the samples was used for first dimension separation on an Ettan^® ^IPGphor^® ^Isoelectric Focusing Unit (GE Healthcare Life Sciences, Uppsala, Sweden) using rehydrated 13 cm immobiline strip (GE Healthcare Bio-Sciences, Uppsala, Sweden) and co-run with internal protein markers (Bio-Rad, Hercules, CA, USA). After first dimensional separation, the gel strip was soaked sequentially in equilibrium buffer pH 6.8 made up of 6 M urea, 30%(w/v) glycerol, 2%(w/v) SDS, 0.05 M Tris-HCl, 2%(w/v) DTT for 30 min and then in 2% iodoacetamide for 30 min. Second dimension separation in 10% SDS gel (18 × 16 cm) was carried out in an ISO-DALT apparatus (Hoefer Scientific Instruments, Amersham Biosciences, San Francisco, CA, USA) operated at a constant current of 60 mA. A Tris-tricine dissociating buffer system was applied. The gel was preserved sequentially in a mixture comprising 40% ethanol and 10% acetic acid, followed by 30% ethanol and stained with silver nitrate. Molecular masses and pI values of the protein spots were determined by co-running with standard markers (MBI Fermantas, Vilnius, Lithuania).

### Image analysis and in-gel digestion

Protein spots of interest were destained with 30 mM potassium ferricyanide and 100 mM sodium thiosulfate, washed with 50% acetonitrile (ACN)/25 mM ammonium bicarbonate at pH 8.0 and soaked in 100% ACN. The gel spots were then dried in a SpeedVac evaporator (UniEquip, Martinsried, Germany), rehydrated in cold trypsin (Promega, Madison, WI, USA) at a concentration of 15 mg/ml (w/v) in 25 mM ammonium bicarbonate, pH 8.0 and incubated at 37°C for 16-24 h. The peptide was then extracted with 50% ACN/5% trifluoroacetic acid (TFA) and reconstituted in 50% ACN/0.1% TFA. The reconstituted extract (0.5 μl) was then mixed with 0.5 μl of fresh cyano-matrix made up of a saturated solution of cyano-4-hydroxy-cinnamic acid (CHCA) in 50% ACN and dispensed on a MALDI plate. The protein sample was analyzed in a TOF mass spectrometer (Voyager-DE PRO Biospectrometry, Applied Biosystems, Foster City, CA, USA). For database search, known contamination peaks such as products of autoproteolysis and keratin were purged before a protein mass fingerprint search was performed on the MASCOT software (Matrix Science, London, UK) in the NCBI database. Up to one missed tryptic cleavage was considered and a mass accuracy of 500 ppm was applied for all tryptic-mass searches. Protein identification was confirmed by using the MS-Fit software http://prospector.ucsf.edu. Several proteins of interest were identified and GDF-8 was selected for further investigation.

### Confirmation of GDF-8 in 2-D gel

Uterine fluid protein separated in 2-D gel as described above was electroblotted onto nitrocellulose membrane. The membrane was blocked with 5% (w/v) non-fat milk (Bio-Rad Laboratories, Hercules, CA, USA) for 2 h prior to interacting with goat polyclonal anti-GDF-8 antibody (1:500) (Santa Cruz Biotechnology, Santa Cruz, CA, USA) at 4°C overnight. After washing with PBS-T (PBS with 0.05% v/v Tween20), the membrane was incubated with HRP-conjugated donkey anti-goat antibody (1:1000) (Santa Cruz Biotechnology, Santa Cruz, CA, USA) and visualized by applying the ECL Plus kit (GE Healthcare, Bio-Sciences, Uppsala, Sweden).

### Molecular cloning of hamster GDF-8

GDF-8 mRNA in hamster uterus was amplified with *Taq *polymerase (GE Healthcare Bio-Sciences, Uppsala, Sweden) and identified by 2% agarose gel electrophoresis. The primer sequences of GDF-8 were: forward: GTGGATGGAAAACCCAAATG; and reverse: TGGTCCTGGGAAGGTTACAG. The 342 bp product was excised and purified with high pure PCR product purification kit (Roche, Mannheim, Germany). The purified PCR product was cloned by using TOPO TA cloning kit dual promoter (Invitrogen, Carlsbad, CA, USA) according to manufacturer's instructions. After transformation, single colonies were purified with blue/white selection. The plasmid was purified with HighPure plasmid purification kit (Invitrogen, Carlsbad, CA, USA) and sequenced by the Genomic Centre of the University of Hong Kong. The GDF-8 sequence was used to synthesize riboprobe for ISH and design probe for real-time PCR.

### *In situ *hybridization

To synthesize digoxigenin (DIG)-labeled GDF-8 riboprobe for ISH, plasmid containing a 302 bp cDNA fragment was linearized with ApaI or PstI, extracted with phenol/chloroform and precipitated with ethanol. The linearized plasmid served as the template for DIG labeling with either T7 or SP6 RNA polymerase in combination with a DIG-RNA labeling kit (Roche, Mannheim, Germany). Deparaffinized sections of the uterus were treated with proteinase K for 15 min and prehybridized in hybridization buffer made up with 4 × SSC (Research Organics, Inc.; Cleveland, OH, USA), 1 × Denhardt's solution, 10 mM DTT, 1 mg/ml yeast tRNA, 10% dextran sulfate and 40% deionized formamide at 37°C for 1 h. The sections were hybridized with DIG-labeled riboprobe specific for GDF-8 (10 ng/ml) at 48°C overnight in a hybridization buffer that contained 1 mg/ml denatured herring sperm DNA (Roche, Mannheim, Germany). After hybridization, the sections were treated with RNase and rinsed in a series of SSC. Incubation with anti-DIG-alkaline phosphatase antibody followed and reaction signals were visualized with NBT/BCIP (Roche, Mannheim, Germany). Sections for negative control were hybridized with the sense probe.

### Immunohistochemistry

Deparaffinized sections of uterine horn were subjected to quenching with 3% hydrogen peroxide in methanol and antigen retrieval in 10 mM sodium citrate (pH 6) at 60°C for 40 min. This was followed by blocking with 5% bovine serum albumin for 30 min and a series of incubations first with goat polyclonal anti-GDF-8 antibody (1:500) (Santa Cruz Biotechnology Inc, Santa Cruz, CA, USA) at 4°C overnight, and then biotinylated anti-goat antibody (1:500) for 2 h. GDF-8 immunoreactivity was visualized by applying the avidinbiotin-peroxidase complex (ABC, Vector Laboratories, Inc.; CA, USA) coupled with diaminobenzidine (DAB; Zymed Laboratories, Inc.; South San Francisco, CA, USA).

### Real-time PCR

cDNA of uterus and endometrium obtained by reversed transcription was used for real time PCR of GDF-8. A 20 μl mixture was prepared according to the manufacturer's manual. It contained 10 μl Taqman^® ^universal PCR master mix (Applied Biosystems, Foster City, CA, USA), 4 μl sterile water, 1 μl 20× Taqman probe of the target gene (Applied Biosystems, Foster City, CA, USA) and 5 μl cDNA. The mixture was subjected to PCR in a light cycler (ABI 7700, Applied Biosystems, Foster City, CA, USA) with conditions recommended by the manufacturer. Upon completion, the PCR data were analyzed by ABI SDS Version 2.1. The cycle threshold (C_T_) was used to compute the fold of change of gene expression at specific time points according to the 2^-ΔΔC^_T _method [14]. Sequences of primers and probes of target genes are given in Table 1.

**Table 1:** Sequences of primers and probes of target genes

Target gene	Sequence
*β-actin*	Forward: GTACCACTGGCATTGTGATGGA Reverse: GAGAGCGTAGCCCTCATAGATG Probe: CAGTGTGGGTGACCCC
*gdf8*	Forward: TGTGCTGGCAGTCCTTTCC Reverse: GAGAAAGGCCAGTACGAATGCT Probe: CACACCTGGGTTCTCT

### Enzyme-linked immunoabsorbent assay for active GDF-8

The expression of active GDF-8 in uterus during pregnancy was quantified with ELISA. Three hundred microgram of uterine protein dissolved in carbonate-bicarbonate buffer (pH 7.4) fortified with 15 mM Na_2_CO_3_, 35 mM NaHCO_3_, 3 mM NaN_3 _was dispensed into the wells of a 96-well plate and left at 4°C overnight. The plate was then washed with PBS-T, blocked with 3% bovine serum albumin (BSA) at 4°C overnight and then immunoreacted overnight with anti-myostatin (1:150; Chemicon, Temecula, CA, USA) at 4°C. This was followed by probing with alkaline phosphatase-conjugated donkey anti-rabbit antibody (1:1000) at 37°C for 1 h. After thorough washing with PBST, 100 ul nitrophenyl-phosphate (NPP) at 2 mg/m1 in a substrate buffer made up of 0.125 mM NaCO_3 _and 1 mM MgCl_2 _was added to each well and incubated at 37°C for 30 min. The reaction was stopped with 10 μl 1N NaOH. The amount of active GDF-8 in the uterus was determined at OD415 with a Microplate Reader (Model 680, Bio-Rad Laboratories, Hercules, CA, USA).

### Myometrial smooth muscle cell (MSMC) culture and quantification

To study the effect of GDF-8 on myometrial smooth muscle cell (MSMC), a primary culture was set up with procedures modified from Shynlova et al. [15]. All chemicals except BSA were purchased from Gibco of Auckland in New Zealand. Uterine horns from a 6-week-old female hamster were blotted free of blood, slit longitudinally, washed and placed in warm Hank's Balanced Salt Solution (HBSS1, pH 7.4) which contained 0.908 g/l MgSO_4_, 0.185 g/l CaCl_2_, 25 mM HEPES, and Penicillin-Streptomycin (P/S). The endometrial layer was gently teased off with fine forceps. The myometrium was washed in HBSS2 (HBSS1 without Mg^2+ ^and Ca^2+^), trimmed into small pieces and disintegrated with shaking in a solution made up of 1 mg/ml collagenase type II, 0.15 mg/ml DNase I, 0.1 mg/ml soybean trypsin inhibitor, 10% Fetal Bovine Serum (FBS) and 1 mg/ml BSA in HBSS2 at 37°C for 1 h. The cells were dispersed by passing through a 40 μm cell strainer. Enzyme digestion was repeated at 4°C overnight. Dissociated cells were precipitated by centrifugation at 1200 g for 10 min. The pellet was washed with HBSS2 that contained 10% FBS and re-suspended in Dulbacco's Mixed Eagle's Medium - Formula 12 (DF12) supplemented with 10% charcoal treated FBS, 1 μM estradiol and 20 μM progesterone at a concentration of 2.5 × 10^4 ^cells per ml. Five hundred microlitres of the cell suspension was dispensed into each well of a 4-well plate (Nunc A/S, Roskilde, Denmark) and incubated for 24 h at 37°C. The heat-sterilized coverslips in the wells had been cleaned, soaked in 0.2N HCl for 30 min and degreased in acetone overnight. Recombinant GDF-8 peptide (R&D, Minneapolis, MN, USA) was added to the cells at a concentration of 5 μg/ml in phenol-red free Dulbacco's Mixed Eagle Medium (DMEM) supplemented with 10% charcoal-treated FBS and 100 U/ml P/S for 60 h or 72 h. Upon completion of treatment, cells were washed with PBS, fixed in 4% paraformaldehyde, permeabilized with 0.1% Triton X-100 and blocked with 3% BSA. Cells were labelled with mouse monoclonal anti-α-smooth muscle actin (SMA) antibody (1:200) (Calbiochem, San Diego, CA, USA) and goat polyclonal anti-PCNA (1:200) (Santa Cruz Biotechnology Inc, Santa Cruz, CA, USA) at 4°C overnight, followed by incubation in sequence for 1 h with FITC-conjugated anti-mouse antibody (1:200) (Jackson ImmunoResearch Laboratory, West Groove, PA, USA) and Alexa Flour 555 conjugated anti-goat antibody (1:200) (Invitrogen, Carlsbad, CA, USA). Nuclei were stained with 6-diamidino-2-phenylindole (DAPI) contained in the UltraCruz™ Mounting Medium (Santa Cruz Biotechnology Inc, Santa Cruz, CA, USA). For negative control, the primary antibodies were omitted. Slides were examined with fluorescence microscope and imaged at 20×. Nine to ten frames were randomly taken for each coverslip from which 995-1923 cells were counted with the Metamorph (version 5.0, Molecular Devices Corporation, PA, USA) or Neurolucidar (Microbrightfield, Colchester, VT, USA) imaging systems. Total numbers of DAPI-stained cells, SMA-positive cells and PCNA cum SMA-positive cells were scored from which the percentages of SMA- and proliferating SMA-labeled cells were calculated.

### Endometrial epithelial cell (EEC) culture and quantification

The procedure for EEC culture was modified from McCormack and Glasser [16]. Uterine horns from 6-week old female hamster were blotted, slit longitudinally and washed in PBS free of Mg^2+ ^and Ca^2+^. They were incubated with shaking firstly in a mixture of trypsin (0.5%, type III from bovine pancreas) and pancreatin (2.5%) dissolved in PBS free of Mg^2+ ^and Ca^2+ ^at 4°C overnight, and then at 20°C for 1 h in HBSS2 fortified with 10% FCS. The medium which contained the EEC was collected. This process was repeated to harvest more ECC. The pooled medium was centrifuged at 500 g for 5 min and the cell pellet was re-suspended in the same medium. After the cell clumps settled by gravity, the supernatant was removed and the process was repeated until the supernatant became clear. The EEC preparation so obtained was free of glandular epithelium, stroma, blood cells and myometrial cells when inspected under the microscope at 400×. The EEC were then pelleted by centrifugation, re-suspended in complete medium made up of DMED/F12, 2.5% FCS, 2.5% Nu Serum, 1 μM estradiol, 20 μM progesterone, 15 mM HEPES, and 1% P/S at 100 U/ml and seeded at a density of 1200 cells/mm^2 ^onto Engelbreth Holm Swarm tumor matrix (Matrigel) coated coverslips in a 24-well plate (BD Biosciences, San Jose, CA, USA). After 24 h, unattached cells were removed and the medium was changed daily for 2 days. GDF-8 at a final concentration of 5 μg/ml was added and the EEC was incubated for a further 36 h. The cells were then fixed in 4% PFA and immunoreacted with mouse anti-keratin antibody and goat anti-PCNA antibody (both at 1:200, Santa Cruz Biotechnology Inc., Santa Cruz, CA, USA) to identify proliferating EEC as described for MSMC. The respective secondary antibodies were Alexa Flour 488 conjugated anti-mouse (1:150) and Alexa Flour 555 conjugated anti-goat at 1:100) (Invitrogen, Carlsbad, CA, USA). Cells that were positive for DAPI, keratin (EEC) and keratin cum PCNA (proliferating EEC) were scored and processed as described for MSMC.

### ELISA for LIF in cultured media

The amount of LIF released into the EEC culture medium was determined as described for active GDF-8. The primary antibody used was goat polyclonal anti-LIF (1:100, Santa Cruz Biotechnology Inc., Santa Cruz, CA, USA) and the secondary antibody donkey anti-goat antibody (1:1000) conjugated to alkaline phosphatase (Jackson ImmunoResearch Laboratory, West Groove, PA, USA). Intensity of immunoreactivity was revealed with nitrophneyl-phosphate and measured at OD415 (Model 680 ELISA Reader, Bio-Rad Laboratories, Hercules, CA, USA).

### Recovery and culture of embryos

Pregnant female hamsters were euthanized at 72 h*pc*. The uterine horns were excised and flushed to recover embryos which were cultured for 48 h with/without 0.75 μg/ml recombinant mouse GDF-8 (R&D Systems, Inc., Minneapolis, USA) in HECM-2h (hatching medium). The hatching medium contained 116.5 mM NaCl, 3.2 mM KCl, 2.0 mM CaCl_2_, 0.5 mM MgCl_2_.6H_2_O, 25 mM NaHCO_3_, 10 mM sodium lactate, 0.25 mM sodium pyruvate, 1 mM glutamine, 0.1 mg/ml polyvinyl alcohol, 100 units/ml Penicillin-Streptomycin (Invitrogen, Carlsbad, CA, USA), 0.001 g/ml phenol red, 0.5 mM succinate, 0.1 mM alanine, 0.1 mM asparagine, 0.1 mM aspartate, 0.1 mM glutamate, 0.1 mM glycine, 0.1 mM proline, 0.1 mM serine, 0.003 mM panthothenate, 0.005 mM choline chloride, 0.003 mM inositol and 3 mg/ml BSA.

Blastocysts were cultured in a 15 μl droplet of hatching medium overlaid with 1.5 ml mineral oil in a 35 × 10 mm^2 ^tissue culture dish (Falcon, Becton Dickinson Labware, NJ, USA) at 37°C in an atmosphere of 5% CO_2 _balance air. After 24 and 48 h, embryos were digitally-imaged (Miticam 2300, Motic China Group Co. Ltd, Xiamen, China) for measurement of area and perimeter with a digitizing program (Motic Images Plus 2.0, Motic China Group Co. Ltd, Xiamen, China). The number of hatched embryos was counted at 48 h in culture.

### Blastocyst attachment

After 48 h in culture, hatched blastocysts were transferred to HECM-2h medium containing 10% fetal bovine serum (Invitrogen, Carlsbad, CA, USA). They were cultured for a further 24 h under the same condition. The number of attached embryos was counted on day 3 of culture. Images of attached embryos were captured for measurement of area/perimeter as described in the last section.

### Differential staining of inner cell mass (ICM) and trophectoderm (TE) of cultured blastocysts

The differential staining of ICM and TE was modified from Handyside and Hunter [17]. Blastocysts at 72 h.*pc *were flushed from excised uterine horns and cultured for 24 h in hatching medium with and without GDF-8. The zona pellucida of cultured blastocysts were removed by acid Tyrode's solution and incubated in HECM-2h for 15-30 min. The zona-free blastocysts were treated with 1:5 diluted anti-hamster whole serum for 15 min at 37°C, washed in the medium and incubated in 1:5 diluted guinea pig complement serum together with 25 μg/ml propidium iodide (PI) for 20 min at 37°C or until all TE cells were stained with PI. After rinsing in PBS, the blastocysts were stained with Hoechst 333258 (25 μg/ml) and mounted in glycerol. The cells of ICM and TE which respectively appeared pink and blue when excited by UV were counted with a fluorescent microscope (ST-18; Carl Zeiss, Oberkochen, Germany).

### Statistical analysis

GDF-8 data obtained by real time PCR and Western blotting at designated days of pregnancy were compared with value for the day of estrous by Student's t-test. To determine the effect of the presence of embryos on active GDF-8 level in the uterus, one way ANOVA was used to analyse data across all time points. The percentages of proliferating MSMC and EEC with and without GDF-8 treatment were compared by Student's t-test. Student's t-test was also used to compare LIF released into the medium of EEC cultures with and without exogenous GDF-8. Student's t-test and χ^2^test were used to determine if GDF-8 had an effect on the number of embryos, TE cells and ICM cells in culture. The level of significance for all statistical analyses was set at P < 0.05.

## Results

### Identification of proteins of interest in uterine fluid and confirmation of GDF-8 in uterine fluid

**Figure 1:**
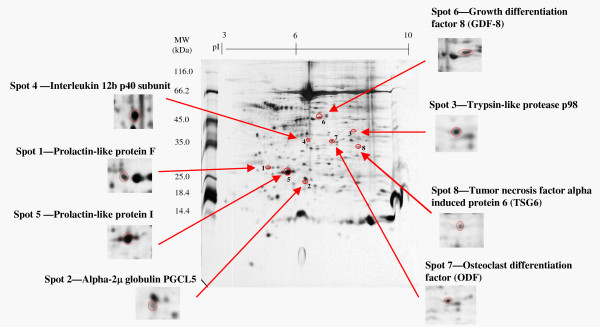
**2D separation of proteins in hamster 72 h *post coital *uterine fluid**. Proteins in uterine fluid were separated by 2D-electrophoresis (Number of animals = 9). The gel was stained with silver nitrate and spots picked, digested with trypsin and analyzed by MALDI-TOF. Eight protein spots of interest were identified (marked in red circle and enlarged in inserts).

Figure 1 shows eight proteins of interest from the hamster 72 h*pc *uterine fluid. Table 2 summarizes the MASCOT score and observed physical parameters of the identified spots. Only GDF-8 was selected for further study because its functional significance in the uterus had never been investigated.

**Table 2:** Summary of proteins identified by MALDI-TOF.

**Spot No**.	Protein Identity	MOWSE Score	Matched No./Total No. (%)	**Accession No**.	Observed MW (×10^3^)/pI	Theoretical MW (×10^3^)/pI
1	Prolactin-like protein F	3.67	6/39 (15)	11968116	28/4.6	29/4.6
2	Alpha-2 μglobulin PGCL5	1045	11/62 (17)	22219456	22/6.1	21/6.1
3	Trypsin-like protease p98	2.41e^4^	14/125 (11)	31542447M	40/8.3	42/8.5
4	Interleukin 12b p40 subunit	4772	15/108 (13)	6652948M	36/6.1	38/6.1
5	Prolactin-like protein I	720	10/78 (12)	13385390	25/5.2	26/5.2
6	Growth-differentiation factor-8 (GDF-8)	3.50e^4^	20/91 (21)	6754752	45/6.3	43/6.6
7	Osteoclast differentiation factor (ODF)	2038	8/70 (11)	4127270	35/7.7	35/78
8	Tumor necrosis factor-induced protein 6 (TSG6)	1.7e^0.8^	25/136 (18)	6678379	33/7.4	31/7.6

Nine proteins of interest in hamster 72 h *post coital *uterine fluid were identified. Information was obtained from on-line database Protein Prospector 4.0.5 provided by University of California, San Francisco http://prospector.ucsf.edu/.

**Figure 2:**
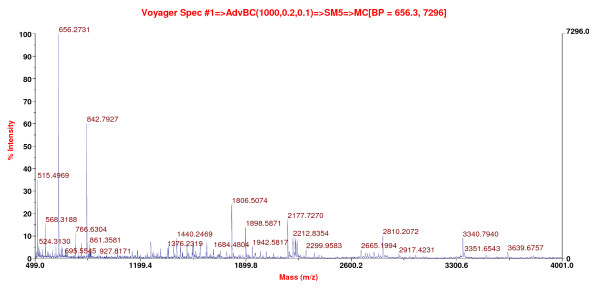
**Representative spectrum of GDF-8 obtained from MALDI-TOF analysis**. The protein spots were picked from hamster 72 h *post coital *uterine fluid 2-D gels. Each protein spot was digested by trypsin overnight, mixed with matrix and placed on sample plate for analysis. Each peak on the spectrum represents a single digested peptide, with its mass annotated).

**Figure 3:**
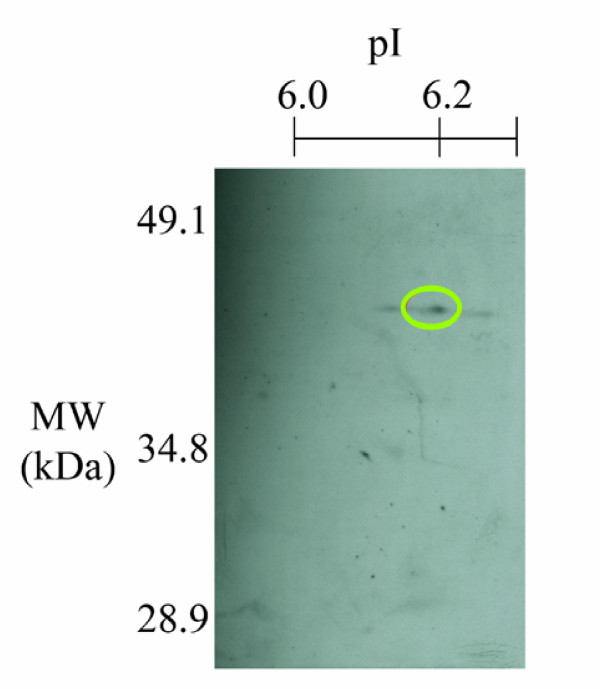
**Confirmation of GDF-8 in hamster uterine fluid by western blotting**. Proteins in 72 h *post coital *uterine fluid were separated by 2D-electrophoresis and transblotted onto nitrocellulose membrane. GDF-8 (green circle) was identified by probing with goat polyclonal anti-GDF-8 antibody and visualized with ECL Plus kit).

Figure 2 displays the raw spectrum of GDF-8 from MALDI--TOF. (For the raw spectra of other proteins, see Additional file 1; Figure S1.) GDF-8 in the hamster uterus had a molecular size of 45 kDa and a pI of 6.7. The identity of GDF-8 was confirmed by immunoblotting. Uterine fluid was separated by 2D gel electrophoresis, transblotted onto nitrocellulose membrane and probed with anti-GDF-8 antibody; a spot at the same location confirmed the presence of GDF-8 (Figure 3).

### Molecular cloning of hamster GDF-8

**Figure 4:**
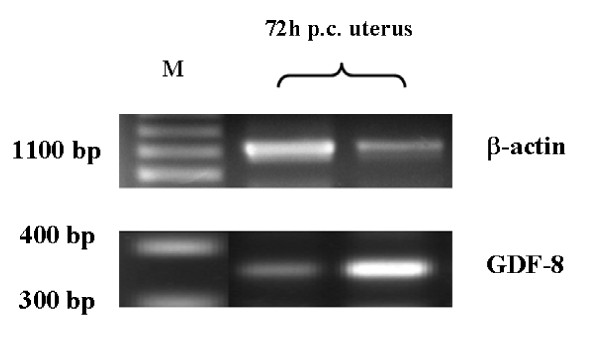
**Gene expression of GDF-8 in hamster 72 h *p*ost coital uterine tissue**. GDF-8 mRNA was amplified with uterine RNA as template. The 342 bp PCR product was cloned and sequenced to unveil the hamster GDF-8 sequence).

**Figure 5:**
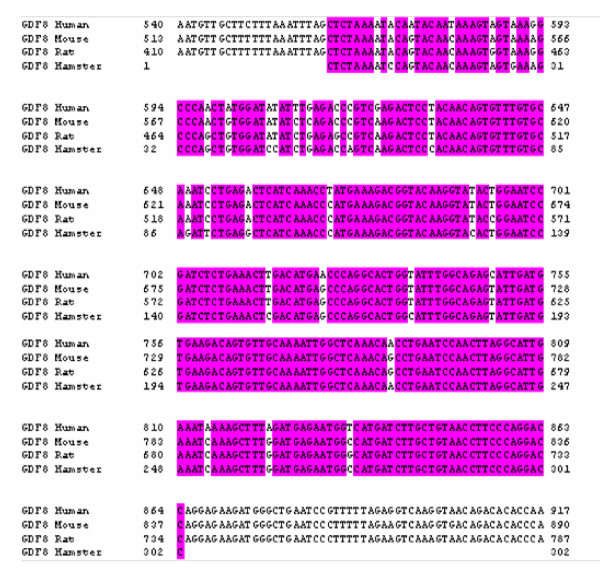
**Homology of hamster GDF-8 sequence with human, rat and mouse sequences**. The hamster GDF-8 mRNA was cloned, sequenced and aligned against human, mouse and rat sequences. They shared over 90% homology).

Primers designed according to homologous regions of GDF-8 in mouse and rats were used to produce a 342 bp amplicon which was cloned and sequenced in hamster uterine tissues (Figure 4). The hamster sequence (excluding primer regions) was found to share respectively 94%, 94% and 92% homology with the mouse, rat and human (Figure 5) which implied that the cloned region was a conserved region of GDF-8 in these species.

### Estimation of uterine GDF-8 mRNA and peptide during pregnancy

**Figure 6:**
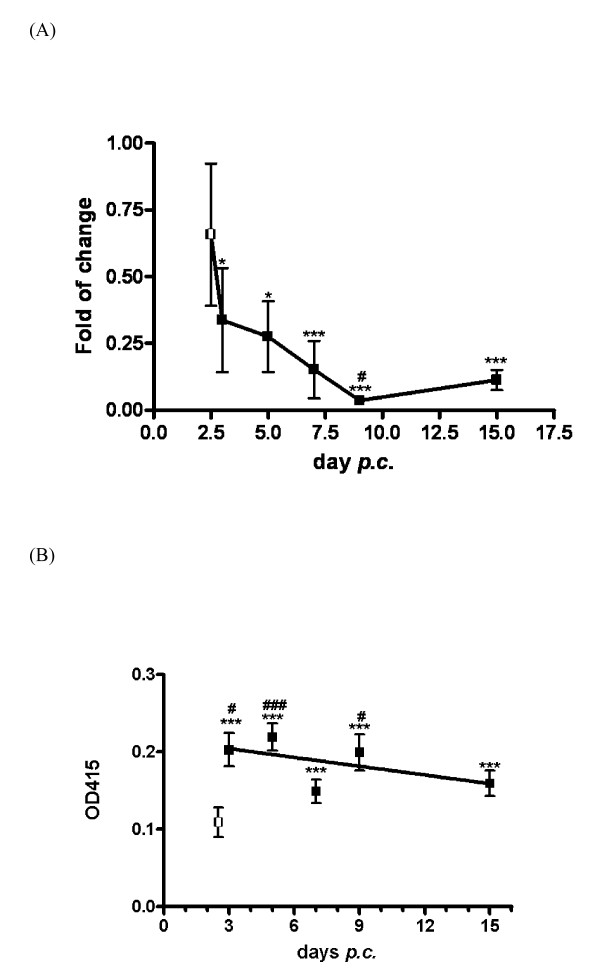
**Temporal profile of GDF-8 in hamster uterus during pregnancy: (A) mRNA expression and (B) cleaved form of the protein**. Total RNA from uterus collected at designated time points *post coitus *was extracted, reversely-transcribed and subjected to real time PCR for amplification of GDF-8. Data were expressed as mean ± S.E.M. To determine how pregnancy affected GDF-8 expression, data from pregnant uterus was compared with non-pregnant uterus at day of estrous (D0) by Student's t-test (*P < 0.05; ***P < 0.01) (Number of animals: D0:6; D2.5:4, D3:4, D5:3, D7:5, D9:5, D15:4). There was a significant reduction in GDF-8 mRNA from D3 onwards. At D2.5 (open squares) the uterus was free of embryos. From D3 onwards (closed squares), embryos were in the uterus. To find out if the presence of embryos affected GDF-8 mRNA expression, data from D3 to D15 were compared with that of D2.5 by one-way ANOVA and a significant decrease was noted only in the D9 uterus ^#^*P *< 0.05). (B) Total uterine proteins at various *post coital *time points were extracted to determine the amount of active GDF-8 peptide by ELISA. Likewise, data were expressed as mean ± S.E.M. Data from pregnant uteri (closed squares) were compared with that of non-pregnant uterus at *estrus *(D0, open squares) by Student's t-test (***P < 0.01) (Number of animals at each time point =3). The amount of active GDF-8 in pregnant uterus was significantly higher than that of non-pregnant uterus. One-way ANOVA was used to determine the effect of the presence of embryos in uterus on active GDF-8 level. The presence of embryo at D3, D5 and D9 has significantly higher level of active GDF-8 (#P < 0.05; ###P < 0.01 compared with D2.5).

Figure 6A illustrates changes of GDF-8 mRNA during the course of pregnancy; data were expressed as fold change relative to an arbitrary baseline at estrus. GDF-8 mRNA dropped significantly from D3 and stabilized at a significantly lower level from D9 onwards. Since hamster embryo normally crossed the oviduct-uterus boundary at D3; data from D3 to D15 was compared with that from D2.5 to find out if the presence of embryos had any effect on uterine GDF-8 mRNA level. A significant reduction was registered at D9. As shown in Figure 6B, the amount of functionally active (cleaved) form of GDF-8 peptide was significantly higher during pregnancy, being highest at D3 when the embryo reached the uterus (*P *< 0.01 at D3, D5 and D9 compared with D2.5).

### Localization of GDF-8 mRNA and peptide in the uterus

**Figure 7:**
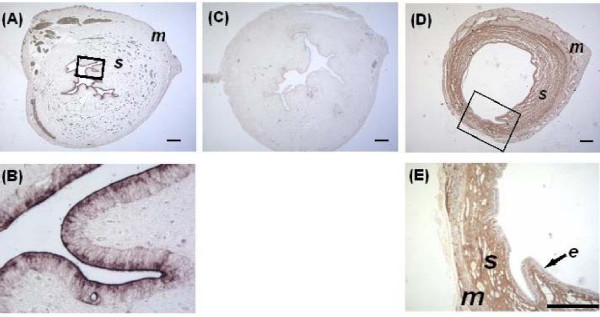
**Localization of GDF-8 transcripts by in situ hybridization (A and B) and peptide by immunohistochemistry (C and D) in the hamster 72 h *p.c*. uterus**. (A) Low magnification of uterine cross section to show that GDF-8 mRNA was confined to endometrial epithelium. (B) Insert magnified to show intense signals in epithelial cells. (C) Absence of signals in negative control of ISH with the sense probe. (D and E) Immunostaining localized GDF-8 peptide to the endometrial stroma (s), but not the myometrium (m) and epithelium (e) (Number of animals =3). Bar = 100 μm).

*In situ *hybridization localized GDF-8 transcripts to the luminal epithelium only (Figure 7A and 7B) while no signal was obtained when the sense probe was used (Figure 7C). Intense immunoreactivity to GDF-8 antibody was observed in cells of the stroma (Figure 7D and 7E) but not the epithelium and myometrium. We suggest that GDF-8 was synthesized in the epithelium but the protein was either released into the uterine fluid or accumulated in the stroma.

### GDF-8 slowed down proliferation of myometrial smooth muscle cells (MSMC)

**Figure 8:**
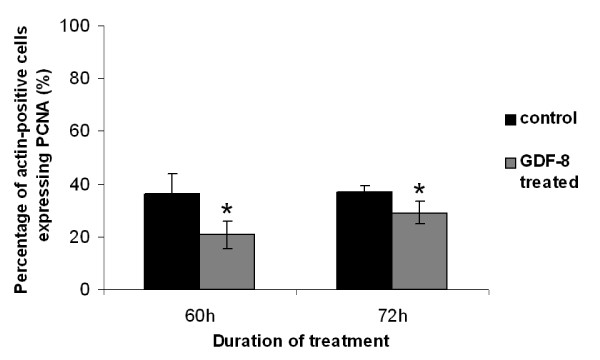
**Effect of GDF-8 on proliferation of hamster myometrial smooth muscle cells (MSMC) in primary culture**. GDF-8 (5 μg/ml) suppressed the proliferation of MSMC. The percentage of proliferating MSMC was compared with the control by Student's t-test (*P < 0.05).

About 64.14 ± 0.04% of cells in the MSMC primary culture were positive for the antibody to α-smooth muscle actin (SMA). These cells also expressed activin receptor type II (ACVR2, data not shown). Administration of GDF-8 at 5 μg/μl for 60 h or 72 h did not change the number of SMA-positive cells but did significantly reduced the number of proliferating cells (*P *< 0.05) (Figure 8). So GDF-8 curtailed proliferation of myometrial cells.

### GDF-8 slowed down proliferation and differentiation of endometrial epithelial cells (EEC) and reduced their LIF secretion

**Figure 9:**
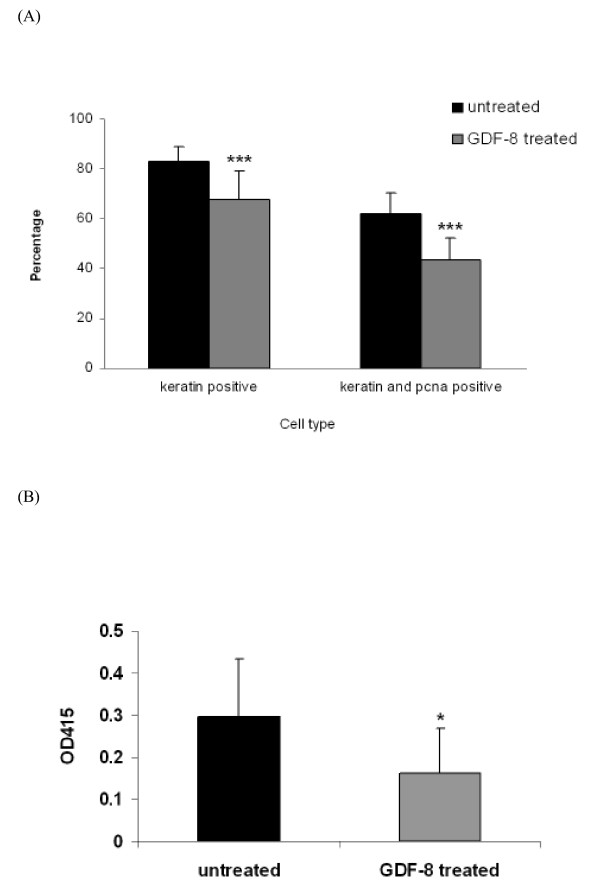
**Effect of GDF-8 on hamster endometrial epithelial cells (EEC) in primary culture**. (A) EEC in primary culture were treated with GDF-8 (5 μg/ml) for 36 h. The percentages of EEC and proliferating EEC were compared with untreated cells by Student's t-test (***P < 0.001). (B) LIF secretion by EEC into the culture medium was determined by ELISA. Data from GDF-8 treated samples was compared with untreated ones by Student's t-test (*P < 0.05). All experiments were repeated four times).

Our preliminary data revealed that EEC also expressed ACVR2 (data not shown), so a primary culture of EEC was set up to investigate the effect of GDF-8 on EEC. EEC was identified by its marker, keratin and the purity of culture was 82.85 ± 6.03. When treated with GDF-8 (5 μg/μl) for 36 h, the numbers of EEC and proliferating EEC both decreased (*P *< 0.001) (Figure 9A). This suggests that GDF-8 exerted a negative effect on proliferation and differentiation of EEC. Figure 9B shows that LIF in the culture medium was significantly lowered by exogenous GDF-8. This could be due to fewer EEC in the culture or a direct effect of GDF-8 on EEC release of LIF.

### GDF-8 stimulated trophectoderm cell proliferation of preimplantation embryos and promoted hatching

The presence and peaking of GDF-8 in the uterus at D3 of pregnancy implied a possible trophic effect on the implanting embryo [12]. To test this, hamster blastocysts were put in culture and treated with GDF-8 (0.75 μg/ml) for 24 h or 48 h. After 24 h incubation, blastocytes were significantly larger as reflected by area and perimeter (Table 3) and this effect sustained as incubation continued to 48 h. The significant increase in total cell number of treated blastocytes was attributed solely to proliferation in the trophectoderm (TE). Cells in the inner cell mass (ICM) remained unchanged. GDF-8 however, reduced the attachment area and perimeter by over 40% (p < 0.001) and 30% (p < 0.01) respectively. These data suggested that GDF-8 modulated the implantation process.

**Table 3:** Effects of GDF-8 on hamster blastocysts in culture.

***Treatment***	**Control**	**GDF-8 (0.75 μg/ml)**
No. of Embryos (at 72 h*pc*)	148	53
Area of embryo image (μm2)	5639.8 ± 125.4	7901.1 ± 317.0***
Perimeter of embryo image (μm)	266.1 ± 2.8	311.8 ± 6.5***
		
*24 h in culture*		
No. of embryos reaching late/expanded blastocyst	82	43
No. of trophectoderm (TE) cells	26.9 ± 1.2	37.6 ± 1.4***
No. of Inner Cell Mass (ICM) cells	15.3 ± 0.7	16.9 ± 0.7
TE/ICM	2.1 ± 0.1	2.4 ± 1.1
Total cell number	42.2 ± 1.3	54.5 ± 1.5***
		
*48 h in culture*		
No. of embryos	70	50
Area (μm2)	5951.6 ± 218.7	9911.1 ± 648.6***
Perimeter (μm)	280.1 ± 4.8	355.9 ± 10.8***
		
*Hatching*		
% embryos hatched (hatched/total No.)	56.9(70/123)	94.3(50/53)++
		
*Attachment*		
No. of embryos	69	50
Area (μm2)	21612 ± 1668	12109 ± 1036***
Perimeter (μm)	603.3 ± 36.1	424.7 ± 21.1**

Number of embryos, TE and ICM without treatment were compared with that of treated embryos with Student's t-test; *P < 0.05; **P < 0.01; ***P < 0.001 compared with control; ++P < 0.001 compared with control using χ^2 ^test

## Discussion

Structural transformation and functioning of the pregnant uterus are largely controlled by steroid hormones, and fine-tuned by signalling molecules, growth factors, immune modulators and adhesion factors. Using proteomic techniques, we profiled some peptides present in the 72 h*pc *uterine fluid of the golden hamster. Several proteins were subjected to further study and they included growth factors, immunomodulators and hormone-related proteins. We selected GDF-8 for functional study because of its potential importance in female reproduction.

GDF-8 is normally associated with development of skeletal muscles [7]. Its presence in the non-pregnant uterus of the human [18] and rat [10] has been reported. It may play a regulatory role in follicular development of bovine and [19] murine species [20]. Other GDF isoforms have been found in the female reproductive system. They include GDF-9 that regulates folliculogenesis and steroidogenesis in ovine and bovine ovaries [19,21,22]; GDF-10 even though present in both pregnant and non-pregnant uterus, appears to be redundant [23]; GDF-5 and GDF-11 are also found in the human endometrium during the secretary phase [18]. Other than these, information on functional significance of GDF is wanting.

In this study, we identified GDF-8 and explored its possible role in pregnancy. We detected by *in situ *hybridization GDF-8 mRNA in endometrial epithelium. GDF-8 protein was identified in uterine fluid by electrophoresis and confirmed by Western blotting. Immunohistochemistry localized its accumulation in endometrial stroma. Its receptor ACVR2 has been localized in all cell types in the uterus of the sheep and pig [24,25]. When we treated primary cultures of myometrial cells (MSMC) and epithelial cells (ECC) with the active GDF-8 peptide, we observed slowing down of proliferation of both cell types which is in line with the well-established anti-proliferative action of GDF-8 on skeletal muscle cells [26,27]. In the EEC primary culture, we noticed that GDF-8 treatment is associated with a reduction of LIF in the medium. This can be a consequence of fewer EEC in culture or a negative effect on release of LIF by EEC. LIF is one of the key inflammatory modulators for implantation [28]. Implantation can be prevented by blocking the action of LIF with its peglyated antagonist [29]. So it is reasonable to suggest that directly or indirectly, GDF-8 may immunomodulate the uterine microenvironment. By real time PCR, we found that GDF-8 gene expression level declined as pregnancy progressed, to reach its nadir during late pregnancy. The amount of active form of GDF-8 peptide also declined. This suggests that GDF-8 is more active in early pregnancy. In our hamster model, the timing is in phase with implantation of embryo when cell division in the uterus is vigorous; thus highlighting the importance of GDF-8 in fine-tuning receptivity of the endometrium.

As for the embryo, GDF-8 is embryotrophic (Table 3). After 24 h in culture with GDF-8, the embryo became larger (area and perimeter when flattened) and its cell number increased significantly. A breakdown into ICM and TE cells by differential staining showed that the stimulatory effect was limited to the TE only. Enlargement of the blastocoel could also imply an action of GDF-8 on the basolateral Na^+^-K^+^ATPase system that mediates the coupled transport of water across the TE [30]. Interestingly, culture of embryos in GDF-8 for 48 h hastened hatching but discouraged attachment which could lead to poorer implantation. To explain this dilemma, we propose that GDF-8 could impair expression of the embryonic implantation serine proteinase (ISP1) gene [31]. All in all, our present results together with the reported finding that GDF-8 could perturb glucose metabolism in human placenta [32], suggest a possible role for GDF-8 in placental development and function.

It has been suggested that GDF-8 released into the uterine environment is beneficial to the developing conceptus because GDF-8 may prevent excessive muscle growth [18]. Evidence to support such a view comes from the fact that administration of soluble form of ACVR2B enhances embryo muscular growth [8]; also a dam which has been immunized with GDF-8 gives birth to offspring with altered composition of crude protein and fat in the body [20]. Apart from muscle, GDF-8 could restrict osteogenesis [33] and promote neurite outgrowth of retinal ganglion cells [34]. As for its origin, GDF-8 is said to be a placental product [32], and in this investigation, we recognized the uterus as the source of GDF-8. It remains to be clarified if the conceptus also secretes GDF-8.

## Conclusion

We report here the identification of GDF-8 in the mammalian pregnant uterus. We noticed that the quantity of GDF-8 mRNA and active peptide both declined as pregnancy progressed, possibly to the advantage of the conceptus for not restricting its growth. Uterine GDF-8 mRNA was localized to luminal epithelial cells but the protein accumulated in endometrial stroma to act on the epithelial cells and myometrial smooth muscle cells in a paracrine manner. With regard to the embryo, the action of GDF-8 is mixed. It stimulated proliferation of TC cells and hatching but discouraged attachment. The latter could possibly be due to disturbance to embryonic implantation protease expression or attenuation of LIF production by endometrial epithelialium. In view of its multi-faceted action on the uterus and embryo, it will be useful to explore the full potential of GDF-8 as a non-hormonal contraceptive and to ascertain its unfavourable side effects in reproductive toxicology because GDF-8 is regarded as a novel therapeutic target for certain metabolic disorders [35].

## Competing interests

The authors have no monetary interest in any chemicals or materials used and are not related to any of the companies that produce them.

## Authors' contributions

CLW worked on hand-mating of hamster, real time PCR, ELISA and drafted the manuscript. YYH carried out primary culture of endometrial epithelial and smooth muscle cells and immunocytochemistry study. WKH participated in proteomics study, immunohistochemistry, molecular cloning and sequence alignment. HKP assisted in hand-mating of hamster, immunohistochemistry, molecular cloning and sequence alignment. PLC was responsible for embryo culture and differential staining of ICM and TE. PHC coordinated the project. PHC and WSO are respectively principal and co-investigators of the research and holders of the grants. All authors read and approved the final manuscript.

## Supplementary Material

Additional file 1 **Representative spectra of other protein spots obtained from MALDI-TOF analysis**. The protein spots were picked from hamster 72 h *post coital *uterine fluid 2-D gels. Each protein spot was digested by trypsin overnight, mixed with matrix and placed on sample plate for analysis. Each peak on the spectrum represents a single digested peptide, with its mass annotated. Click here for file
