# Supplementary figures and images for: Growth-differentiation factor-8 (GDF-8) in the uterus: its identification and functional significance in the golden hamster
Source: Reprod Biol Endocrinol. 2009 Nov 25;7:134. doi: 10.1186/1477-7827-7-134 (PMC2790456; doi:10.1186/1477-7827-7-134)

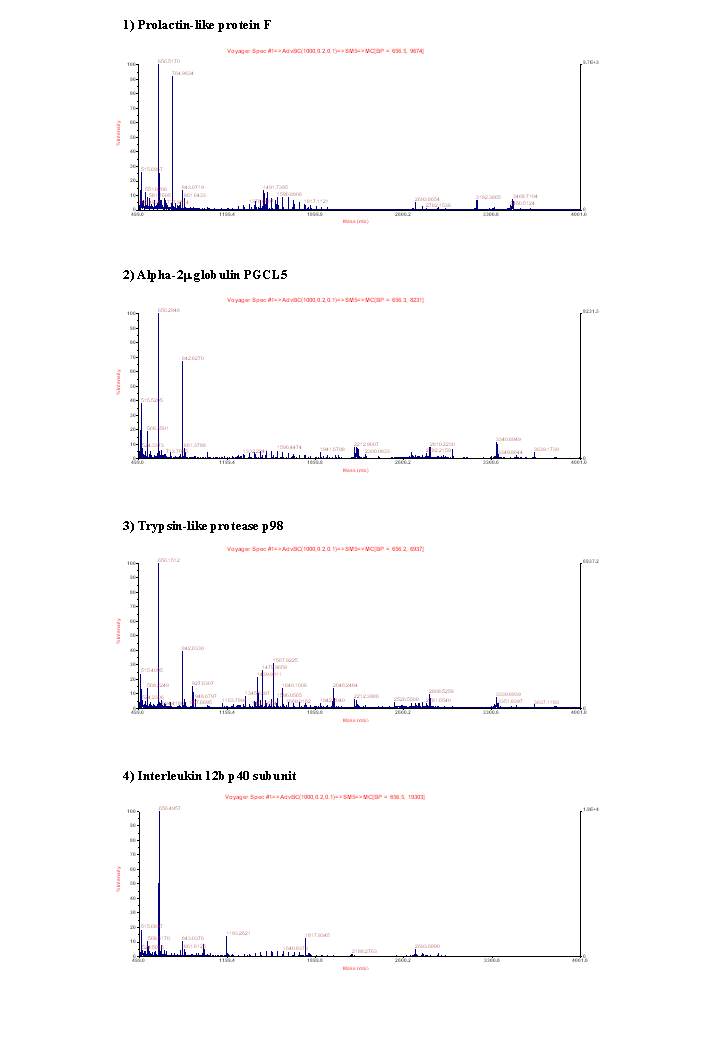


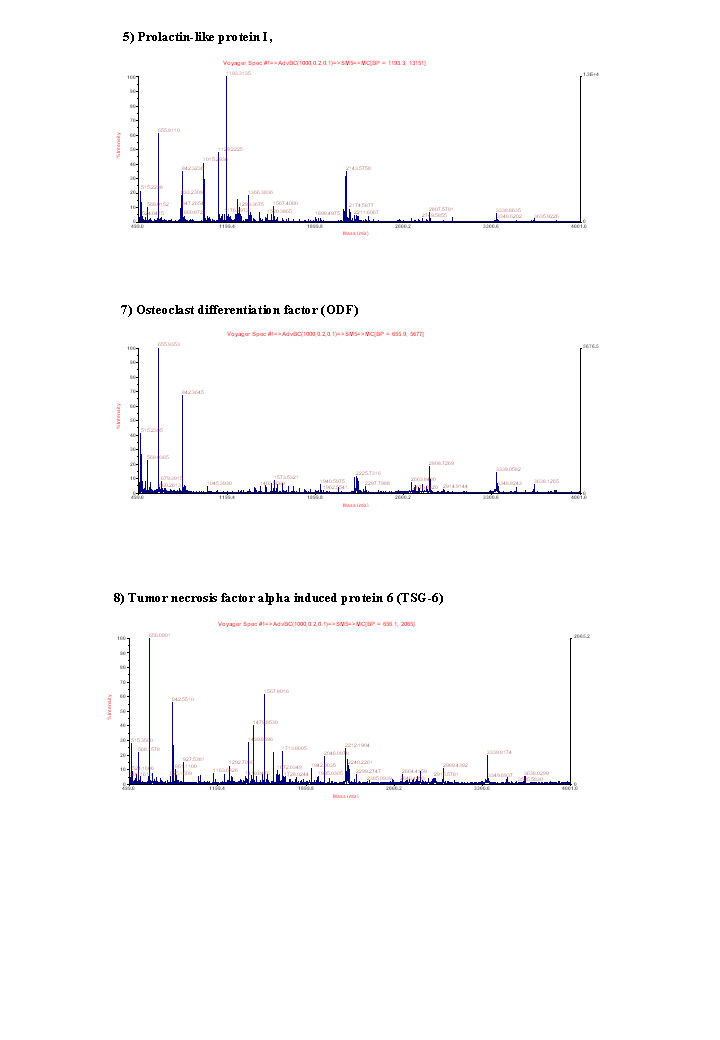

Supplement: Additional file 1 — Representative spectra of other protein spots obtained from MALDI-TOF analysis. The protein spots were picked from hamster 72 h post coital uterine fluid 2-D gels. Each protein spot was digested by trypsin overnight, mixed with matrix and placed on sample plate for analysis. Each peak on the spectrum represents a single digested peptide, with its mass annotated. [file 1477-7827-7-134-S1.doc]
